# Low *Usutu virus* seroprevalence in four zoological gardens in central Europe

**DOI:** 10.1186/1746-6148-9-153

**Published:** 2013-08-06

**Authors:** Nicola Buchebner, Wolfgang Zenker, Christian Wenker, Hanspeter W Steinmetz, Endre Sós, Helga Lussy, Norbert Nowotny

**Affiliations:** 1 Viral Zoonoses, Emerging and Vector-Borne Infections Group, Institute of Virology, University of Veterinary Medicine Vienna, Vienna, Austria; 2 Vienna Zoo Schönbrunn, Vienna, Austria; 3 Basel Zoo, Basel, Switzerland; 4 Clinic for Zoo Animals, Exotic Pets and Wildlife, Vetsuisse Faculty, University of Zurich, Zurich, Switzerland; 5 Budapest Zoo, Budapest, Hungary; 6 Department of Microbiology and Immunology, College of Medicine and Health Sciences, Sultan Qaboos University, Muscat, Oman; 7 Kleintierklinik Breitensee, Vienna, Austria; 8 Tierarztpraxis Neuwiesen, Uster, Switzerland; 9 Knies Kinderzoo, Rapperswil, Switzerland

## Abstract

**Background:**

*Usutu virus* (USUV), a mosquito-borne flavivirus of the *Japanese encephalitis virus* antigenic group, caused bird die-offs in Austria, Hungary and Switzerland between 2001 and 2009. While the zoological gardens of Vienna and Zurich recorded USUV-associated mortality in different species of birds during this period, incidences in Budapest were limited to areas outside the zoo, and in the greater Basel area avian mortality due to USUV infection was not observed at all. The objectives of this investigation were to gain insight into USUV infection dynamics in captive birds in zoos with varying degrees of virus exposure and to study differences in susceptibility to USUV of different species of birds.

**Results:**

372 bird sera were collected between October 2006 and August 2007. The samples were tested in parallel by hemagglutination inhibition (HI) and 90% plaque reduction neutralization tests (PRNT-90). 8.75%, 5.3% and 6.59% of birds in the zoos of Vienna, Zurich and Basel, respectively, showed USUV-specific antibodies by PRNT-90. No antibodies to USUV were detected in birds of the Budapest zoo. The order *Strigiformes* (owls) exhibited the highest USUV-seroprevalence, compared to other orders of birds.

**Conclusions:**

USUV seems not to pose an imminent threat to zoo bird populations in central Europe at the moment. Depending on a variety of especially environmental factors, however, this may change at any time in the (near) future, as experienced with West Nile virus (WNV). It is therefore strongly suggested to continue with combined WNV and USUV surveillance activities in affected areas.

## Background

*Usutu virus* (USUV) is a mosquito-borne flavivirus (family *Flaviviridae*) of the *Japanese encephalitis virus* antigenic group. It was first isolated from mosquitoes in South Africa in 1959 [1] and named after a river in Swaziland. The virus had never been associated with fatal disease in animals or humans. In summer 2001, however, USUV emerged in and around Vienna, Austria, causing mortality among wild birds, particularly Eurasian blackbirds (*Turdus merula*) [2]. The virus continued to cause avian deaths in Austria during subsequent years [3,4], extending its range of circulation to other adjacent Austrian federal states. USUV-associated bird die-offs were also recorded among wild and zoo birds in neighbouring countries: in Hungary, starting in 2005 [5], in Italy from 2006 onwards [6], in Switzerland also since 2006 [7], in Germany since 2011 [8], and in the Czech Republic also since 2011 [9]. A recently conducted retrospective analysis of archived tissue samples originating from a bird die-off in the Tuscany region (Italy) in 1996, however, revealed the presence of USUV in Europe long before 2001 [10]. In 2009, two human cases of severe neuroinvasive USUV infections were reported from Italy [11,12]; both patients, however, had suffered from severe comorbidities.

Birds kept in zoological gardens have frequently been victims of USUV infection: one of the first outbreaks of USUV associated bird mortality in Europe was recorded in 2001 in the Vienna zoo, where five great grey owls (*Strix nebulosa*) succumbed to the infection [2]. In summer 2006, an USUV associated bird die-off occurred in the Zurich zoo [7] and its adjacencies. The involved birds were members of the orders *Passeriformes* (blackbirds and sparrows) and *Strigiformes* (owls). Mortality was also recorded in captive owls in Northern Italy between 2006 and 2008 [6]. No obvious USUV associated bird mortality was observed in the Basel zoo and in the Budapest zoo, although in and around the city of Budapest a number of Eurasian blackbirds succumbed due to USUV infection [5].

This study’s objectives were i) to test several species of birds, kept in four different zoological gardens in three countries, for the presence of USUV-specific antibodies; two of the selected zoos (Vienna and Zurich) had experienced USUV associated bird mortalities, while the other two (Basel and Budapest) did not report clinical USUV cases; ii) to compare the USUV antibody prevalence in the four zoological gardens; iii) to identify bird species with a higher susceptibility to USUV infection; and iv) to compare a hemagglutination inhibition (HI) test with a plaque reduction neutralization test 90% (PRNT-90) for the detection of USUV antibodies.

## Results

### HI test

A total of 96 out of 372 (25.8%) sera showed HA inhibition against USUV antigen in the HI test, 43 out of 132 (32.58%) sera from Zurich, 40 out of 91 (43.96%) from Basel, 11 out of 80 (13.75%) from Vienna, and only 2 out of 68 (2.94%) from Budapest. The titers ranged from 1:20 to 1:2560 with an arithmetic mean of 1:142.5.

### PRNT-90

In contrast to the HI test results, only 20 out of 372 birds tested positive for USUV-specific antibodies by PRNT-90. Seven positives were detected in Zurich (5.30%) and Vienna (8.75%), respectively, and 6 in Basel (6.59%). The sera obtained from birds in the Budapest zoo proved all USUV antibody negative in PRNT-90. The PRNT titers ranged from 1:20 to 1:640 with an arithmetic mean of 1:143 (Table 1). The 20 sera positive by PRNT-90 USUV were re-tested by a West Nile virus (WNV) PRNT-90; only the two sera with USUV PRNT titers of 1:640 reacted positive against WNV, each with a titer of 1:20.

**Table 1:** Summary of the study results

**Order**	**Family**	**Genus**	**Species (English name)**	**Species (Latin name)**	**Number tested**	**PRNT antibody titers to USUV**						
						***<*20 neg**	**20**	**40**	**80**	**160**	**320**	**640**
Rheiformes	Rheidae	Rhea	Greater Rhea	Rhea americana	1	1	0	0	0	0	0	0
Casuariiformes	Casuariidae	Casuarius	Double-wattled Cassowary	Casuarius casuarius	1	1	0	0	0	0	0	0
Sphenisciformes	Spheniscidae	Spheniscus	**Humboldt Penguin**	**Spheniscus humboldti**	19	18	0	0	0	**1**^**b**^	0	0
			Black-footed Penguin	Spheniscus demersus	1	1	0	0	0	0	0	0
Pelecaniformes	Pelecanidae	Pelecanus	Dalmatian Pelecan	Pelecanus crispus	5	5	0	0	0	0	0	0
			White Pelecan	Pelecanus onocrotatus	3	3	0	0	0	0	0	0
Ciconiiformes	Phoenicopteridae	Phoenicopterus	**Greater Flamingo**	**Phoenicopterus ruber**	109	103	0	**2**^**c**^	**2**^**c**^	**2**^**c**^	0	0
			Lesser Flamingo	Phoenicopterus minor	5	5	0	0	0	0	0	0
	Ciconiidae	Ciconia	**White Stork**	**Ciconia ciconia**	23	22	0	0	**1**^**a**^	0	0	0
		Leptoptilos	**Marabou Stork**	**Leptoptilos crumeriiferus**	2	1	**1**^**b**^	0	0	0	0	0
	Threskiornithidae	Geronticus	Hermit Ibis	Geronticus eremita	2	2	0	0	0	0	0	0
	Ardeidae	Nycticorax	Black-crowned Night Heron	Nycticorax nycticorax	1	1	0	0	0	0	0	0
Anseriformes	Anatidae	Branta	**Red**-**brested Goose**	**Branta ruficollis**	9	8	0	0	**1**^**b**^	0	0	0
			Hawaiian Goose	Branta sandvicensis	2	2	0	0	0	0	0	0
		Anser	Lesser White-fronted Goose	Anser erythropus	4	4	0	0	0	0	0	0
			Greylag Goose	Anser anser	1	1	0	0	0	0	0	0
			Domestic Goose	Anser anser domestica	2	2	0	0	0	0	0	0
			Bar-headed Goose	Anser indicus	6	6	0	0	0	0	0	0
		Cereopsis	Cereopsis Goose	Cereopsis novaehollandiae	3	3	0	0	0	0	0	0
		Alopochen	Egyptian Goose	Alopochen aegyptiacus	1	1	0	0	0	0	0	0
		Tadorna	**Ruddy Shellduck**	**Tadorna ferruginea**	2	1	0	0	**1**^**b**^	0	0	0
		Chloephaga	Magellan Goose	Chloephaga picta	2	2	0	0	0	0	0	0
		Dendrocygna	Fulvous Whistling Duck	Dendrocygna bicolor	2	2	0	0	0	0	0	0
		Tachyeres	**Steamer Duck**	**Tachyeres pteneres**	3	2	0	0	0	0	0	**1**^**b**^
		Anas	Domestic Duck	Anas platyrhynchos domesticus	4	4	0	0	0	0	0	0
			Mallard	Anas platyrhynchos	2	2	0	0	0	0	0	0
		Aix	Wood Duck	Aix sponsa	1	1	0	0	0	0	0	0
			Mandarin Duck	Aix galericulata	1	1	0	0	0	0	0	0
		Cygnus	Mute Swan	Cygnus olor	1	1	0	0	0	0	0	0
			Tundra Swan	Cygnus columbianus	2	2	0	0	0	0	0	0
Falconiformes	Accipitridae	Neophron	**Egyptian Vulture**	**Neophron percnopterus**	4	3	0	0	**1**^**a**^	0	0	0
		Haliaeetus	White-tailed Eagle	Haliaeetus albicilla	2	2	0	0	0	0	0	0
		Aquila	Imperial Eagle	Aquila heliaca	1	1	0	0	0	0	0	0
		Circaetus	Short-toed Eagle	Circaetus gallicus	2	2	0	0	0	0	0	0
		Buteo	Common Buzzard	Buteo buteo	6	6	0	0	0	0	0	0
	Pandionidae	Pandion	Osprey	Pandion haliaetus	1	1	0	0	0	0	0	0
	Falconidae	Falco	Common Kestrel	Falco tinnunculus	5	5	0	0	0	0	0	0
			Saker Falcon	Falco cherrug	1	1	0	0	0	0	0	0
Galliformes	Phasianidae	Gallus	**Domestic Chicken**	**Gallus gallus domesticus**	60	59	0	0	0	0	0	**1**^**b**^
		Lophophorus	Himalayan Monal	Lophophorus impejanus	1	1	0	0	0	0	0	0
		Pavo	Common Peafowl	Pavo cristatus	9	9	0	0	0	0	0	0
		Tragopan	Satyr Tragopan	Tragopan satyra	1	1	0	0	0	0	0	0
	Cracidae	Crax	Great Curassow	Crax rubra	1	1	0	0	0	0	0	0
	Meleagrididae	Meleagris	Wild Turkey	Meleagris gallopavo	4	4	0	0	0	0	0	0
Gruiformes	Gruidae	Grus	Common Crane	Grus grus	1	1	0	0	0	0	0	0
			Manchurian Crane	Grus japonensis	1	1	0	0	0	0	0	0
		Balearica	Grey Crowned Crane	Balearica regulorum	2	2	0	0	0	0	0	0
	Otididae	Ardeotis	Kori Bustard	Ardeotis kori	1	1	0	0	0	0	0	0
Charadriiformes	Burhinidae	Burhinus	Spotted Dikkop	Burhinus capensis	1	1	0	0	0	0	0	0
Columbiformes	Columbidae	Goura	Western Crowned Pigeon	Goura cristata	2	2	0	0	0	0	0	0
		Columba	Domestic Pigeon	Columba livia domestica	6	6	0	0	0	0	0	0
Psittaciformes	Psittacidae	Amazona	White-fronted Amazon	Amazona albifrons	1	1	0	0	0	0	0	0
		Cyanoliseus	Patagonian Conure	Cyanoliseus patagonus	8	8	0	0	0	0	0	0
		Ara	Green-winged Macaw	Ara chloroptera	1	1	0	0	0	0	0	0
Strigiformes	Strigidae	Bubo	**Eurasian Eagle Owl**	**Bubo bubo**	5	3	0	**1**^**a**^	0	**1**^**a**^	0	0
			**Snowy Owl**	**Bubo scandiacus**	2	1	0	**1**^**a**^	0	0	0	0
		Asio	Long-eared Owl	Asio otus	3	3	0	0	0	0	0	0
		Tyto	Barn Owl	Tyto alba	1	1	0	0	0	0	0	0
		Strix	**Ural Owl**	**Strix uralensis**	4	2	0	**1**^**a**^	**1**^**a**^	0	0	0
Coraciiformes	Halcyonidae	Dacelo	**Laughing Kookaburra**	**Dacelo novaeguineae**	2	1	0	0	0	**1**^**b**^	0	0
	Meropidae	Merops	European Bee-eater	Merops apiaster	10	10	0	0	0	0	0	0
	Coraciidae	Coracias	European Roller	Coracias garrulus	1	1	0	0	0	0	0	0
Passeriformes	Corvidae	Corvus	Rook	Corvus frugilegus	1	1	0	0	0	0	0	0
		Pica	European Magpie	Pica pica	1	1	0	0	0	0	0	0
**Total**					372	352	**1**	**5**	**7**	**5**	0	**2**

Avian species exhibiting Usutu virus-specific neutralizing antibodies are indicated in bold. Code: ^**a**^ – Vienna zoo; ^**b**^ – Zurich zoo; ^**c**^ – Basel zoo; no positives were recorded in the zoo of Budapest.

### Comparison of HI and PRNT results

Nineteen out of 20 PRNT positive sera were also positive by HI test. On the other hand, however, only 19 out of 96 HI positive sera were confirmed by PRNT. All further results refer to the PRNT-90 titers.

### Zurich zoo

In Zurich, USUV-specific antibodies were detected in one marabou stork (*Leptoptilos crumeriiferus*) with a titer of 1:20, one ruddy shellduck (*Tadorna ferruginea*) and one red-breasted goose (*Branta ruficollis*), each with a titer of 1:80, one Humboldt penguin (*Spheniscus humboldti*) and one laughing kookaburra (*Dacelo novaeguineae*), both with a titer of 1:160, one steamer duck (*Tachyeres pteneres*) and one domestic chicken (*Gallus gallus domesticus*), each with a titer of 1:640.

### Basel zoo

All 6 positive samples from the Basel zoo originated from greater flamingos (*Phoenicopterus ruber*). Their titers ranged from 1:40 to 1:160 with an arithmetic mean of 1:93.3.

### Vienna zoo

In Vienna, antibodies were found in one snowy owl (*Bubo scandiacus*) showing a titer of 1:40, two ural owls (*Strix uralensis*) having titers of 1:40 and 1:80, respectively, one white stork (*Ciconia ciconia*) and one Egyptian vulture (*Neophron percnopterus*), each with a titer of 1:80, and two Eurasian eagle owls (*Bubo bubo*) with titers of 1:40 and 1:160, respectively.

### Budapest zoo

No USUV PRNT positive sample was detected in the Budapest zoo. The results of the study are summarized in Table 1.

## Discussion

Birds in zoological gardens seem to be preferred primary targets of emerging mosquito-borne flaviviruses, as it was observed for USUV in the zoos of Vienna [2], Zurich [7], and in a big collection of owls in Milan [6]; also, one of the first recorded WNV outbreaks in America occurred in a zoo [13]. Zoo veterinarians exclude the possibility of importation of infected birds to the zoos due to strict quarantine procedures applied. Thus, two possible explanations for these observations remain: i) the wide variety of avian species kept in zoos with some being vulnerable to a newly introduced mosquito-borne virus, and ii) the awareness of bird mortality is close to 100% in the setting of a zoological garden, while in the wild avian deaths are only recognized in case of mass die-offs.

Besides the first reports on the emergence of USUV in the zoos of the different countries no follow-up studies were performed. The goal of this study was therefore to investigate the USUV antibody prevalence in a variety of avian species in the zoos of Vienna, Zurich, Budapest and Basel, whereas in the zoos of Vienna and Zurich USUV-associated bird mortality had been observed, while no USUV cases were reported from the zoos in Basel and Budapest (but a few cases were identified in the greater Budapest city area).

A study that involves drawing blood from mostly very valuable species of birds is subject to restrictions. The animal ethics committees of the four zoological gardens involved only approved blood draws on two occasions: i) during essential veterinary treatment, and ii) on the occasion of Influenza A (H5N1) vaccinations of the birds. Thus, statistic-driven random sampling was not possible. Nonetheless a total number of 372 birds, belonging to 64 different species, could be included in the study.

In accordance with Meister et al. [14], the HI test proved to be a useful, cheap, host species independent and easy-to-handle screening test. Only one out of 20 sera that proved positive in the ‘gold-standard’ PRNT was negative in the HI test. On the other hand, 77 HI-positive sera must be considered non-specific for USUV, most likely due to the well-known antigenic cross-reactivity of flaviviruses [15,16]. WNV competitive ELISAs may also be used for a combined WNV / USUV / tick-borne encephalitis virus (TBEV) antibody screening, although these assays also show cross-reactions with other flaviviruses and thus the ELISA results must be verified by virus-specific PRNT, too [17]. Although WNV had never been observed in Switzerland and it emerged in Austria not before 2008 [18,19], i.e. 1–2 years after the sera had been collected for the present study, we tested the 20 USUV PRNT-positive sera also by PRNT-90 against WNV; only the two sera with the highest USUV antibody titers (1:640) showed neutralizing activity against WNV at the lowest dilution (1:20), which indicates low-level cross reactivity between these closely related flaviviruses.

The samples collected in the zoo of Zurich were a well-diversified cross section of many species of birds. A rather low percentage (5.3%) of birds exhibited USUV-specific antibodies, with titers ranging between 1:20 and 1:640. The order of *Anseriformes* accounted for almost half of the seropositives. Interestingly, no bird of this order was USUV antigen or -nucleic acid positive during the USUV associated bird die-off in this zoo [7]. The serological results obtained indicate partial susceptibility of this avian order to USUV infection, which, however, did not result in clinical disease or death. The rather subclinical nature of USUV infection in geese was also demonstrated experimentally [20].

The samples obtained in the Vienna zoo also consisted of a wide range of avian species. With 8.75% positive results, Vienna exhibited the highest percentage of USUV-specific antibodies of all zoos studied. The titers varied from 1:40 to 1:160. Despite having the highest percentage of positives, the low to medium level of antibody titers suggests that the Vienna zoo has not been exposed to USUV in the recent past, which is supported by the fact that no USUV associated bird mortalities were recorded there since 2007. The distribution of USUV seropositives within the investigated avian species clearly indicates a higher susceptibility of birds of the order *Strigiformes* to USUV infection. Contrary to *Anseriformes* USUV invades in *Strigiformes* regularly the CNS, with lethal consequences. The high vulnerability of owls for USUV infection was demonstrated in several previous studies [2,6,7,21]. Actually, owls seem to be the second most vulnerable species of birds for the central European strain of USUV next to the Eurasian blackbird (*Turdus merula*).

In the zoo of Basel we did not expect evidence for USUV infection since neither in the zoo nor in the Basel area USUV associated bird mortality had been observed in the past. Surprisingly, however, 6.59% of birds tested positive for USUV antibodies by PRNT with titers ranging from 1:40 to 1:160. All positive birds were greater flamingos (6 out of 80 tested greater flamingos). None of the flamingos, however, had shown overt signs of disease, or had died. Flamingos seem to be in general susceptible to certain flavivirus infections, as evidenced for WNV on the occasion of the emergence of this virus in the U.S. [13]. On the other hand, USUV antibody positive birds in the absence of overt disease resembles the USUV and WNV epidemiology in birds reported for the U.K. by Buckley et al. [22]. Since in the zoo of Basel only very few other avian species could be investigated, no statement can be made regarding the susceptibility to USUV for other species of birds.

In the Budapest zoo 68 birds of 28 species belonging to 11 orders were tested, but none of them exhibited detectable USUV antibodies. USUV associated bird mortality had not been observed in this zoo, in the city of Budapest and its surroundings, however, limited blackbird mortality was attributed to USUV infection [5]. Most likely, the birds in Budapest zoo were not exposed to USUV.

Despite the limitations in sampling it was possible to investigate serum samples of 372 birds, belonging to 64 species, kept in captivity in 4 different zoos in 3 central European countries. This is the first survey for USUV-specific antibodies in mostly exotic species of birds kept in zoos in or close to USUV-endemic areas.

One of the surprising outcomes of this study was a much lower USUV antibody prevalence than expected, which is in contrast to a similar investigation carried out on certain wild bird species [14]. It seems that only a rather small proportion of exotic bird species is susceptible to USUV infection, which is actually good news for zoological gardens.

Summarizing the current literature, the most vulnerable species of birds for the central European strain of USUV remains to be the Eurasian blackbird [2-10,21], followed by different species of owls [2,6,7,21], this study] and the house sparrow [3,7,21]. Besides the above species, single cases of USUV associated mortality were reported from a variety of other songbird species [3,4], especially within the order Passeriformes.

## Conclusions

The present investigations did not reveal high USUV vulnerability of any other major order or species of birds besides those already known. On the other hand, the high susceptibility of owls (*Strigiformes*) to USUV infection is strongly supported by the present study, which actually parallels observations on WNV [23,24]. We also expected the order of *Falconiformes* to be more affected by USUV but did not obtain enough evidence to support this hypothesis.

Based on our study and the absence of converse reports we can conclude that USUV seems not to pose an imminent threat to zoo bird populations in central Europe at the moment. Depending on a variety of especially environmental factors, however, this situation may change at any time in the (near) future, as experienced with WNV. It is therefore strongly suggested to continue the combined WNV and USUV surveillance activities in the affected areas.

## Methods

Bird sera were collected in the zoos of Basel, Budapest, Vienna, and Zurich between October 2006 and August 2007. In total, sera of 372 zoo-birds, belonging to 15 different orders (142 *Ciconiiformes*, 78 *Galliformes*, 48 *Anseriformes*, 22 *Falconiformes*, 20 *Sphenisciformes*, 19 *Columbiformes*, 15 *Strigiformes*, 13 *Coraciiformes*, 10 *Psittaciformes*, 8 *Pelicaniformes*, 5 *Gruiformes*, 2 *Passeriformes*, 1 *Casuariformes*, 1 *Choradriiformes*, 1 *Rheiformes*), 25 families, 51 genera, and 64 species were investigated (Table 1). One hundred and thirty two samples were collected at the zoological garden in Zurich, 80 samples at the zoo in Vienna, 91 specimens at the Basel zoo and 68 samples at the Budapest zoo. In Zurich and Basel blood was taken on the occasion of Influenza A (H5N1) vaccinations, whereas in Vienna and Budapest samples were obtained only from birds that were examined because of injuries or during other investigations. All tested birds except 18 greater flamingos (*Phoenicopterus ruber*) from Basel zoo were older than one year. The majority of investigated birds at Basel zoo were greater flamingos (80 out of 91).

In all cases between 0.3 and 1.0 ml of blood was drawn from the cutaneous ulnar vein, the jugular vein or a vein on the leg. The blood was centrifuged at 2000 × g for 10 min. The serum was separated from the clot and stored at −20°C until investigated. The serum samples were inactivated at 56°C for 30 min before analyses. In order to obtain information about sensitivity and specificity of the easy-to-perform HI test, each serum was submitted to two different assays: an HI test and an USUV-specific PRNT-90, the “gold standard” in flavivirus serology.

### HI test

The HI test was conducted as described by Clarke and Casals [25]. First, non-specific inhibitors were bound and removed by kaolin treatment, followed by an isoagglutinin treatment through adsorption with 1% goose erythrocytes. Then, 25 μl serum was mixed with 25 μl [= 8 hemagglutination (HA) units] of USUV antigen (strain Vienna 2001 [26]) and transferred into U-shaped microtiter plates. The HI titer was defined as the highest serum dilution that caused complete inhibition of erythrocyte agglutination by 8 HA units of viral antigen. A titer of ≥ 1:20 was considered positive.

### PRNT-90

The PRNT was carried out as described by De Madrid and Porterfield [16], modified to a microtechnique [27]. The procedure was run in flat-bottomed, 96-well microplates. The inactivated sera were mixed with 30 μl of virus suspension (USUV strain Vienna 2001 [26]), incubated at 37°C for 60 min and then supplemented with 60 μl of cell suspension (porcine kidney cells in Minimal Essential Medium with 3% fetal calf serum). After an incubation period of 4 hours at 37°C, 120 μl of carboxy-methyl cellulose overlay was added to each well and further incubated at 37°C. After 3 days the fluid was removed and 150 μl of a coloring agent (naphtol blue black solution) was added and incubated for 40 min at room temperature. The PRNT titer was defined as the highest serum dilution with an at least 90% reduction of the number of plaques. A titer of ≥1:20 was regarded positive.

The PRNT-90 against WNV was carried out as described above for USUV, but with WNV strain Eg101 [17].

## Competing interests

None of the authors of this paper has a financial or personal relationship with other people or organizations that could inappropriately influence or bias the content of the paper. The authors declare that they have no competing interests.

## Authors’ contributions

NB carried out all the experimental work and drafted the manuscript. WZ, CW, HWS and ES assisted in the blood draws and provided valuable information on the birds and the zoos, respectively. HL carried out the laboratory tests. NN coordinated and supervised the study and revised the manuscript. All authors read and approved the final manuscript.
